# Tumor-Dependent Cytotoxicity of Novel Benzodiazepinones Across Cancers with Distinct IAP-Associated Aggressiveness

**DOI:** 10.3390/molecules31214016

**Published:** 2026-11-07

**Authors:** Michele Fiore, Dario Lunaccio, Elisa Fagnani, Paolo Cocomazzi, Francesco Bonì, Bruno Tasso, Annalisa Salis, Enrico Millo, Federica Cossu

**Affiliations:** 1 Institute of Biophysics-National Research Council (IBF-CNR) Genoa Unit, via De Marini, 6, 16149 Genoa, Italy; michele.fiore@ibf.cnr.it; 2 Department of Experimental Medicine, Section of Biochemistry, University of Genoa, Viale Benedetto XV 1, 16132 Genoa, Italy; lunacciodario@gmail.com (D.L.); annalisa.salis@unige.it (A.S.); 3 Institute of Biophysics-National Research Council (IBF-CNR) Milan Unit, Via Corti 12, 20133 Milano, Italy; elisafagnani@cnr.it (E.F.); paologiuseppecocomazzi@cnr.it (P.C.); francesco.boni@cnr.it (F.B.); 4 Department of Pharmacy, Section of Medicinal Chemistry, School of Medical and Pharmaceutical Sciences, University of Genoa, Viale Benedetto XV, 3, 16132 Genoa, Italy; bruno.tasso@unige.it

**Keywords:** benzodiazepinones, IAPs, cancer, cytotoxicity, invasiveness, chemoresistance, medicinal chemistry

## Abstract

Inhibitor of Apoptosis Proteins (IAPs) are key regulators of tumor progression, invasiveness, and chemoresistance, and their contribution varies across different tumor types. Here, we evaluated an expanded library of benzodiazepinone derivatives in invasive and chemoresistant human cancer cell lines (MDA-MB-231, BT-549, NCI-H1299, and PC-3), in which IAP-dependent signaling has been implicated to different extents. These compounds were developed from a parental benzodiazepinone previously shown to bind IAPs in vitro using isolated proteins and to target IAPs in MDA-MB-231 cells. The expanded library retained cytotoxic activity beyond MDA-MB-231 cells, extending to BT-549 and NCI-H1299, whereas limited effect was observed in PC-3 cells. Tumor Necrosis Factor (TNFα) co-treatment further enhanced the cytotoxic activity of selected derivatives, although the magnitude of this effect differed among cell lines, suggesting a differential contribution of TNFα/NF-κB (Nuclear Factor kappa-light-chain-enhancer of activated B cells) signaling, potentially involving IAP regulated pathway. Overall, these findings expand the biological profile of this benzodiazepinone scaffold and support its further investigation as a promising lead for the development of therapies against aggressive and chemoresistant cancers.

## 1. Introduction

Inhibitor of apoptosis proteins are key regulators of apoptosis through their direct interaction with caspases, inhibiting their proteolytic activity [1]. Their activity is physiologically counteracted by Smac/DIABLO (Second mitochondria-derived activator of caspases/Direct IAP binding protein with low pI), which binds the baculoviral IAP repeat (BIR) domains of IAPs and promotes apoptosis [2]. Based on this mechanism, small-molecule Smac-mimetics (SMs) were developed as IAP antagonists able to induce cellular IAP (cIAP) degradation and restore apoptotic signaling, mainly through TNFα-dependent mechanisms [3,4]. However, despite their promising anticancer potential, resistance to SMs has been widely described and is often associated with loss of TNFα sensitivity, impaired cytokine production, and increased expression of cIAP2 and other IAP family members that sustain NF-κB signaling and cell survival [5]. Indeed, SMs are particularly effective in combination with TNFα, whereas tumor cells unable to produce this cytokine are frequently refractory to treatment with SMs alone. These limitations have shifted attention toward alternative strategies aimed at targeting residual IAP functions rather than inducing their complete degradation. In particular, the BIR1 domains of cIAP2 and XIAP (X chromosome-linked IAP), involved in the regulation of NF-κB activation through interaction with proteins such as TRAF2 (tumor necrosis factor receptor-associated factor 2) and TAB1 (TGF-beta Activated Kinase 1 (MAP3K7) Binding Protein 1), represent promising targets [6,7]. This approach has led to the identification of novel IAP modulators able to interfere with BIR1-mediated complexes, including **NF023** [8], **FC2**, and benzodiazepinone derivatives, which modulate NF-κB-dependent survival pathways and induce apoptosis [9,10].

The relevance of IAPs as therapeutic targets is particularly evident in aggressive and chemoresistant cancer models, where the role of IAPs has been described with different degrees of significance. MDA-MB-231 triple-negative breast cancer (TNBC) cells represent a highly invasive model sensitive to SMs due to autocrine TNFα production, which promotes a switch from survival to apoptosis or necroptosis upon IAP degradation [11]. In xenograft models, **SM83** treatment reduced tumor growth, correlating with degradation of cIAP1, cIAP2, and XIAP [12]. Moreover, cIAP1 and cIAP2 contribute to MDA-MB-231 invasiveness through regulation of Epidermal Growth Factor Receptor/Snail family transcriptional repressor 2 (EGFR/Snai2), Epithelial–Mesenchymal Transition (EMT), and AKT signaling [13,14]. Conversely, the BT-549 TNBC model, characterized by chemoresistance mainly associated with apoptosis suppression, shows limited sensitivity to SMs as single agents due to impaired TNFα production, although TNFα induction by combination treatments can restore responsiveness [15,16]. Similarly, PC-3 prostate cancer cells, a highly aggressive and chemoresistant model derived from bone metastasis, display correlation between cIAP1/2 and XIAP levels and TNFα sensitivity [17,18]. Accordingly, IAP targeting through Smac mimetics enhances cisplatin-induced apoptosis and inhibits PC-3 cell growth [19,20]. Moreover, NF-κB activation correlates with prostate cancer aggressiveness and poor clinical outcome, while inhibition of NF-κB signaling reduces IAP expression, restores TNFα responsiveness, and limits PC-3 growth and invasiveness [21,22]. In NCI-H1299 non-small cell lung cancer cells, resistance to SM-induced apoptosis has been linked to elevated cIAP2 levels and USP11-mediated stabilization [5,23]. The clinical trajectory of IAP antagonists underscores that IAPs degraders require combination of sensitizing agents (i.e., chemo- or radiotherapy) to induce cell death [24]. Moreover, extensive IAP degradation may carry immune-related liabilities [25,26].

**Scheme 1:**
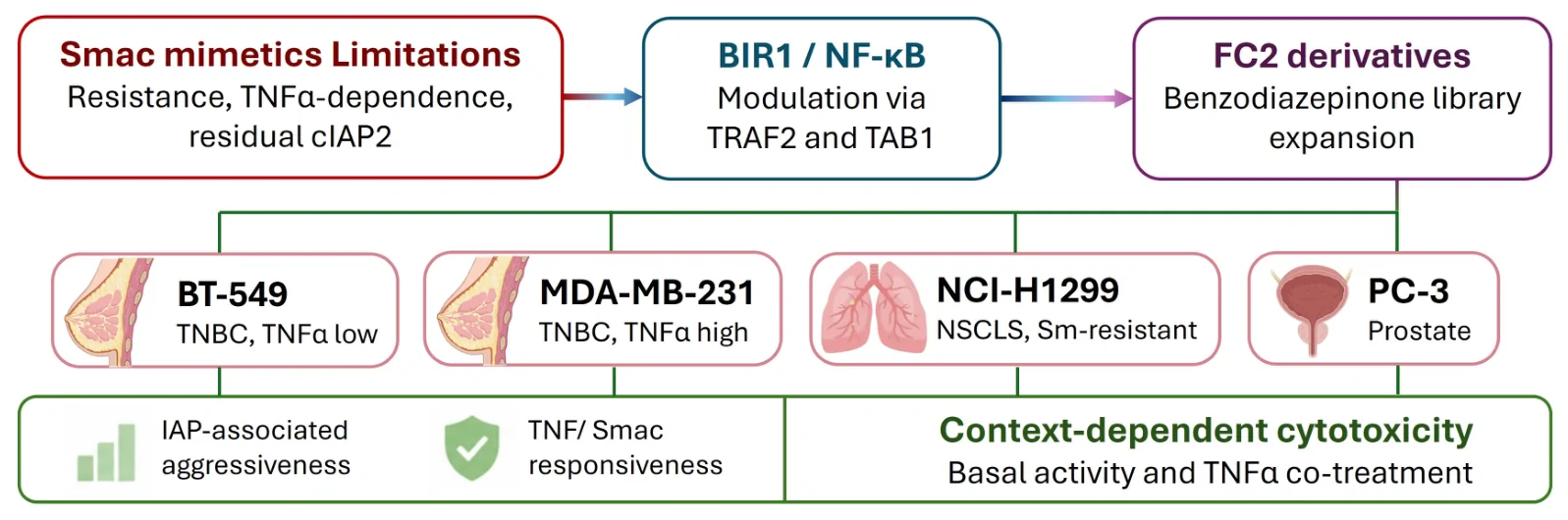
Rationale of the study. The limitations of IAP-antagonists (Smac mimetics), linked to TNFα-dependent resistance and residual cIAP2 expression, have redirected attention toward modulation of the NF-κB signaling and of the IAP complexes mediated by the BIR1 domain. In this framework, FC2-derived benzodiazepinones were evaluated in four aggressive cancer models with different degrees of IAP dependency and TNFα responsiveness, to define their cytotoxicity profile both as single agents and upon TNFα co-treatment.

For these reasons, modulation of IAP-regulated pathways may represent an alternative approach to overcome tumor cell survival mechanisms beyond classical SM-induced degradation. However, whether FC2-derived benzodiazepinones retain their biological activity across tumors characterized by different degrees of IAP dependency and TNFα responsiveness remains unknown. Since these two factors critically influence the efficacy of conventional Smac mimetics, addressing this question is essential to determine whether this scaffold may display a broader therapeutic potential than classical IAP degraders. In this context, benzodiazepinone derivatives derived from the **FC2** scaffold represent promising candidates, as the parental compound was previously shown to target IAPs in vitro and in MDA-MB-231 cells [9,10]. Here, we expanded this chemical library with novel benzodiazepinone derivatives to explore their activity across a broader panel of invasive and chemoresistant cancer models. Compounds were evaluated in MDA-MB-231, BT-549, NCI-H1299, and PC-3 cell lines, selected for their established but differential involvement of IAP-dependent mechanisms (Scheme 1). Accordingly, TNFα co-treatment was included not only to evaluate compound sensitization but also to determine whether differences in TNFα responsiveness contribute to the tumor-specific activity profile of this benzodiazepinone scaffold.

## 2. Results

### 2.1. Chemical Synthesis and Library Expansion

**Figure 1:**
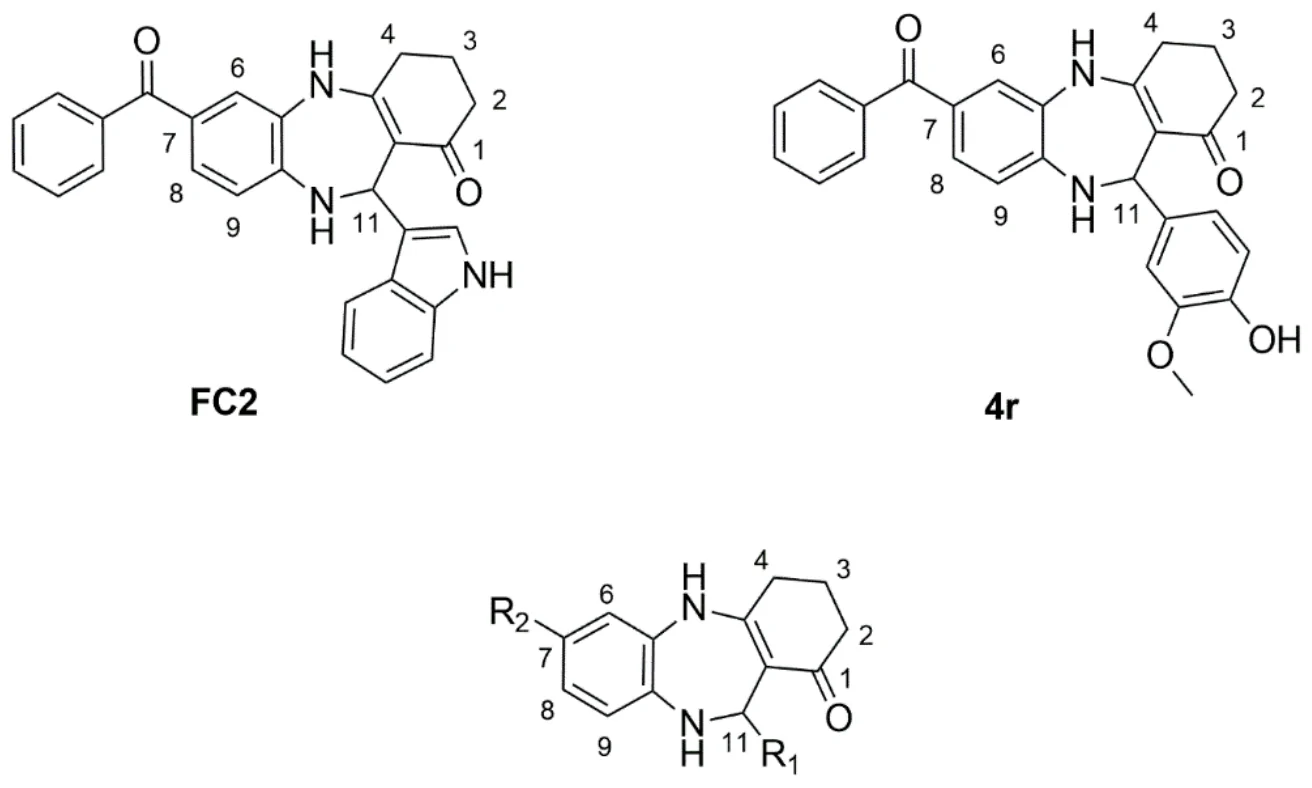
Structure of parental benzodiazepinone hits **FC2** and **4r** and scaffold for the design of novel benzodiazepinone derivatives.

The benzodiazepinone library was expanded starting from the previously identified IAP-modulating scaffold **FC2** and its optimized derivative **4r** [10]. The synthetic strategy was designed to explore additional chemical space around the benzodiazepinone core through modifications at two main positions of the scaffold (C-11 and C-7, namely R_1_ and R_2_), aiming to identify structural features affecting biological activity across different cancer models (Figure 1).

Derivatives bearing modifications at the C-11 (namely, R_1_) position were synthesized according to our previously reported procedure [10]. Briefly, the synthesis involved the condensation of 3,4-diaminobenzophenone derivatives with cyclohexane-1,3-diones in a H_2_O/EtOH (1:1) mixture at T = 80 °C, yielding the corresponding enamine intermediates. Subsequent cyclocondensation with selected aldehydes in the presence of catalytic acetic acid afforded the final 1H-dibenzo [1,4]diazepin-1-one derivatives **1-6** (Figure 1 and Table 1). Complete synthetic procedures and compound characterization data are reported in the Supporting Information.

**Figure 2:**
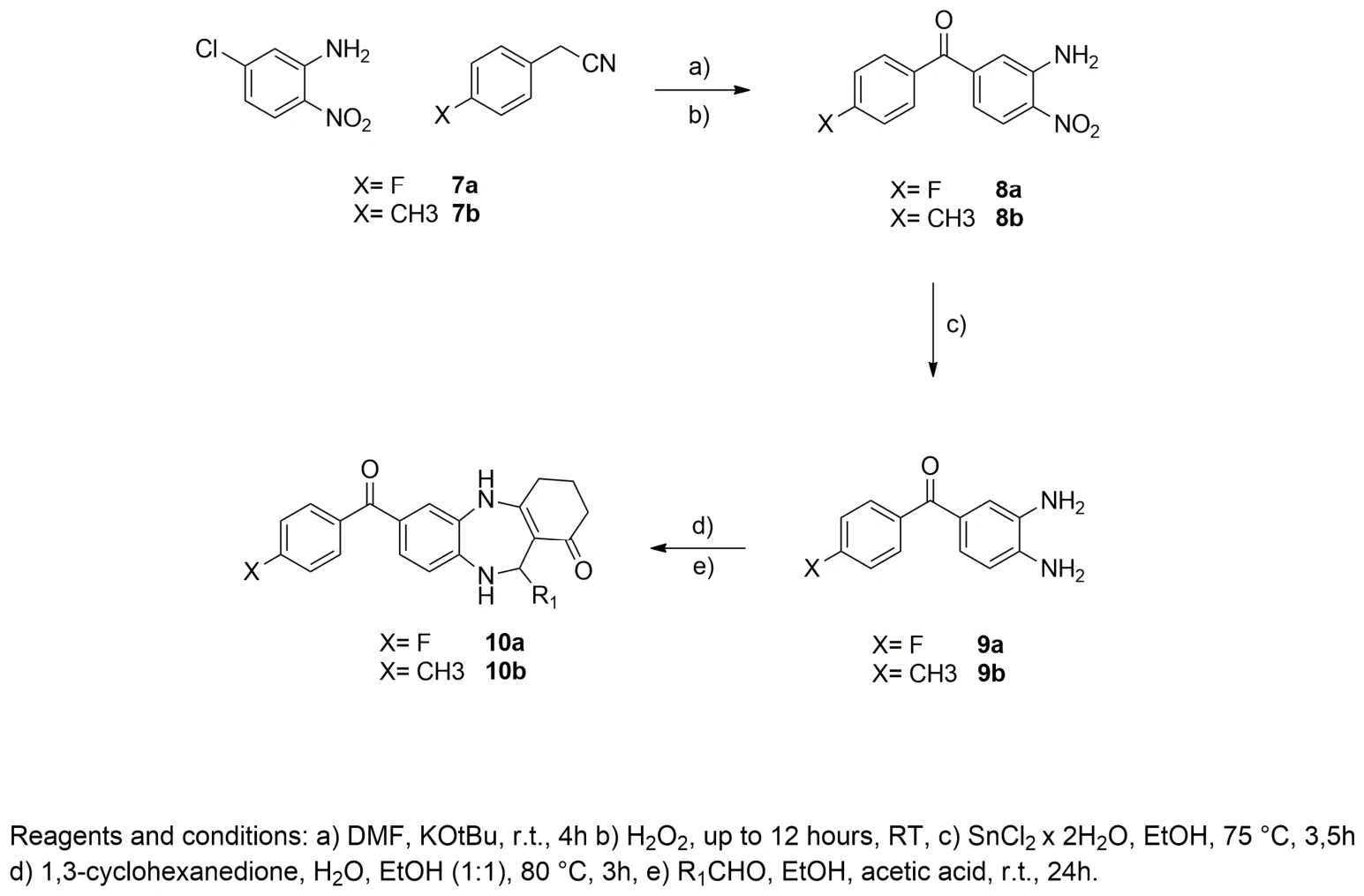
Scheme of the synthesis of the benzodiazepinones **10a** and **10b**.

To expand the chemical diversity at the C-7 (namely, R_2_) position, a modified synthetic approach was developed (Figure 2).

The previously employed Friedel–Crafts acylation strategy represented a limiting step due to poor flexibility toward aromatic substitution patterns and restricted access to differently substituted benzophenone intermediates. Therefore, an alternative nucleophilic aromatic substitution-based route was established. In this approach, substituted phenylacetonitriles **7a**, **7b** reacted with activated 5-chloro-2-nitroaniline to generate nitrile intermediates, which underwent in situ oxidative decyanation using aqueous hydrogen peroxide to provide the corresponding nitrobenzophenone derivatives **8a**, **8b** [27]. Subsequent reduction with SnCl_2_·2H_2_O yielded the required diamino benzophenone intermediates **9a**, **9b**, which were converted into the final benzodiazepinone derivatives **10a**, **10b** following the established condensation/cyclization protocol (Figure 2).

Overall, this synthetic strategy enabled the preparation of a focused library of benzodiazepinone derivatives with increased structural diversity around the parental scaffold.

### 2.2. Cytotoxic Activity Across Tumor Models

The benzodiazepinone library expanded from **FC2** and **4r** was initially evaluated in MDA-MB-231 triple-negative breast cancer (TNBC) cells and NCI-H1299 non-small cell lung cancer cells, two models characterized by invasive phenotypes and established involvement of IAP-dependent survival pathways. The parental compound **4r** was included as a reference molecule, while the newly synthesized derivatives were assessed for their ability to reduce cell viability and compared according to their structural modifications (Table 1).

In MDA-MB-231 cells, most derivatives displayed moderate cytotoxic activity in the low micromolar range, with several compounds retaining or improving the activity of the parental scaffold. Among the R_1_-modified derivatives, **1** showed the strongest activity (EC_50_ = 5.0 ± 1.0 µM), suggesting that increased electron-donating substitution on the aromatic ring may favour activity in this cellular context. Similarly, halogenated derivatives **3**, **4**, and **6** maintained good potency, with EC_50_ values (EC_50_ = 7.1 ± 2.9 µM, EC_50_ = 9.3 ± 1.1 µM, EC_50_ = 5.4 ± 0.8 µM, respectively) comparable to **4r** (EC_50_ = 7.5 ± 1.7 µM). Conversely, modifications introducing increased flexibility or additional substitutions at other positions of the scaffold generally reduced activity, and electronic features appear to contribute for optimal response in MDA-MB-231 cells.

A distinct activity profile emerged in NCI-H1299 cells. Compared with MDA-MB-231, derivatives bearing electron-withdrawing halogen substituents showed markedly enhanced potency, as in the case of compounds **3**, **4**, and **6**, displaying EC_50_ values around 1.2–1.3 µM. Conversely, **1** and **2**, which were among the more active compounds in MDA-MB-231 cells (EC_50_ = 5.0 ± 1.0 µM and EC_50_ = 10.5 ± 1.9 µM), showed limited activity in NCI-H1299 cells (EC_50_ = 42.8 ± 1.9 µM and 47.4 ± 2.8 µM). These differences suggest that the structural determinants governing cytotoxicity may be dependent on tumor context.

To further investigate the biological relevance of this scaffold, the cellular panel was expanded to include BT-549 TNBC cells and PC-3 prostate cancer cells, which were selected because of their aggressive and chemoresistant tumor models and their different reported dependence on IAP-mediated signaling. A single-concentration screening approach (12.5 µM) was applied to evaluate the broader activity profile of representative derivatives.

**Figure 3:**
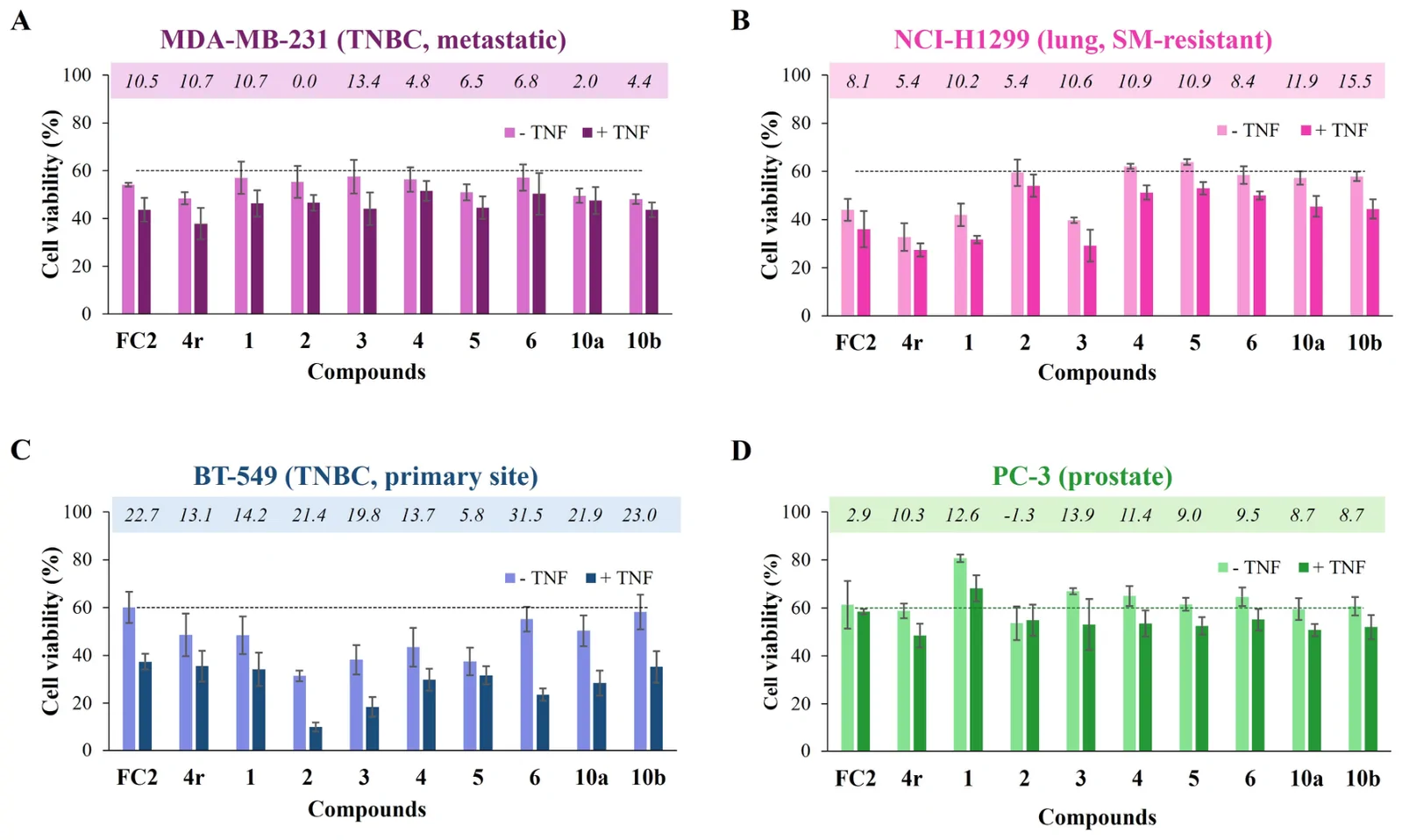
Cell viability of different cancer cell lines ((**A**), MDA-MB-231 (purple); (**B**), NCI-H1299 (pink); (**C**), BT-549 (blue); (**D**), PC-3 (green)) upon 48 h treatment with a fixed concentration (12.5 µM) of benzodiazepinone derivatives. The dashed line indicates the threshold at 60%. The variations in viability upon co-treatment with 50 ng/mL TNFα are reported as percentages in colored boxes (according to the cell-line color code), calculated as the difference between the cell viability values observed in the absence of TNFα and those measured in the presence of the cytokine. Each experimental condition was tested in three replicate wells in at least three independent experiments.

MDA-MB-231 cells (Figure 3A) showed a more homogeneous moderate response, confirming the activity of the scaffold in the parental IAP-related model, while revealing limited improvement through library expansion. The structural optimization mainly affects activity in other cellular contexts. The NCI-H1299 model (Figure 3B) exhibited selective sensitivity toward specific derivatives, particularly **4r** and halogen-containing analogues (**1**, **3**), highlighting the contribution of chemical features to tumor-specific activity.

In the context of TNBC, compared to MDA-MB-231 cells, the BT-549 model (Figure 3C) showed an overall higher sensitivity to benzodiazepinone derivatives, including the parental **4r**, with several compounds reducing viability below the predefined activity threshold (set at 60%). Compared with **FC2** and **4r**, stronger effects were observed for **2**, **3** and **5**, suggesting that despite their resistance to IAP-antagonists as single agents, BT-549 cells remain responsive to selected benzodiazepinone derivatives.

Conversely, PC-3 cells (Figure 3D) represented the least responsive model within the panel. Most compounds at 12.5 µM displayed limited basal cytotoxicity, with viability values generally above the activity threshold. Notably, derivatives highly active in MDA-MB-231 or NCI-H1299 cells, such as **1**, **3** and **10a** (Table 1, Figure 3A,B), showed substantially reduced efficacy in PC-3 cells. These results highlight the importance of tumor-specific biological determinants in controlling compound responsiveness and suggest that IAP/NF-κB dysregulation alone may not be sufficient to predict sensitivity to benzodiazepinones.

### 2.3. TNFα Sensitization and IAP-Related Pathway Relevance

Because IAPs are central regulators of TNFα-dependent survival and NF-κB signaling, the effect of TNFα co-treatment was evaluated across all cellular models. The addition of TNFα (50 ng/mL) revealed distinct sensitization patterns, further supporting the relevance of cellular context in the study of benzodiazepinones’ activity.

In MDA-MB-231 cells, TNFα exposure produced heterogeneous effects among the tested compounds. While several derivatives showed limited changes in EC_50_ values, selected molecules displayed enhanced cytotoxicity. In particular, **10a** showed approximately three-fold increased potency following TNFα treatment (EC_50_ = 4.6 ± 0.8 µM vs. EC_50_ = 16.7 ± 1.0 µM), whereas **4** and **10b** also showed improved activity, although to a lesser extent (EC_50_ = 4.5 ± 1.0 µM vs. EC_50_ = 9.3 ± 1.1 µM and EC_50_ = 8.2 ± 1.3 µM vs. EC_50_ = 17.0 ± 2.8 µM). These observations suggest that specific structural features may influence the ability of compounds to exploit TNFα-dependent apoptotic pathways in this TNBC model.

Analogously, NCI-H1299 cells displayed moderate TNFα-mediated sensitization. Although TNFα enhanced the activity of some derivatives in the single-concentration assay (Figure 3B), EC_50_ analysis revealed limited changes following cytokine treatment. This behaviour is consistent with IAP-dependent resistance to IAP-antagonists (Smac-mimetics, SMs), associated with cIAP2 stabilization and impaired apoptotic signalling [5,23].

A stronger TNFα-dependent effect was observed in BT-549 cells (Figure 3C). Although these cells are poorly responsive to SMs due to reduced endogenous TNFα production, TNFα supplementation markedly enhanced the activity of several benzodiazepinone derivatives. Compounds **2** and **6** showed particularly pronounced reductions in viability following TNFα exposure, indicating that restoration of TNFα signaling may increase susceptibility to derivative-induced cell death.

Finally, PC-3 cells, despite their limited basal sensitivity, showed a moderate but statistically significant decrease in viability for individual compounds following TNFα treatment (Figure 3D), although the effect remained lower than that observed in the other models. This suggests that TNFα signaling can partially overcome intrinsic resistance mechanisms but that additional factors regulate responsiveness in prostate cancer cells.

## 3. Discussion

In this study, we expanded the chemical space of a benzodiazepinone scaffold of a previously identified IAP-engaging NF-κB modulator (**FC2**) through the synthesis and biological evaluation of a focused library of novel derivatives. MDA-MB-231 cells were confirmed to be a suitable primary screening model for the expanded benzodiazepinone library. Interestingly, extending the study to additional aggressive cancer models (BT-549, NCI-H1299, and PC-3) revealed a context-dependent cytotoxic profile for FC2-derived benzodiazepinones, with differential sensitivity across tumor types.

Structural modifications at the R_1_ position suggested distinct cell-dependent structure–activity relationships, with halogen substituents favoring activity in NCI-H1299 cells and methoxy substituents favoring activity in MDA-MB-231 cells. Conversely, increased conformational flexibility or additional modifications of the phenyl moiety generally resulted in reduced activity, highlighting the importance of specific steric and electronic features for optimal biological response. Starting from **4r**, modifications in R_2_ resulted in slightly worse EC_50_ values in MDA-MB-231, while retaining a good profile in the SMs-resistant NCI-H1299. PC-3 cells remained largely refractory to both benzodiazepinone treatment and TNFα-mediated sensitization, whereas the other cancer models exhibited distinct degrees of sensitivity to TNFα, with BT-549 cells exhibiting the most pronounced enhancement of cytotoxicity. These findings demonstrate that the benzodiazepinone scaffold retains cytotoxic activity beyond the parental MDA-MB-231 model and suggest that the tumor contexts and the functional status of TNFα/IAP-regulated signaling could influence the biological response to these compounds.

Although these compounds are structurally derived from **FC2**-related benzodiazepinones previously shown to interact with cIAP2/XIAP BIR domains and modulate IAP-dependent pathways, direct target engagement by the expanded library remains to be established. Future studies will therefore focus on validating IAP binding and elucidating the molecular basis of the observed context-dependent cytotoxicity, including the effects on IAP-regulated signaling complexes and NF-κB activation. In addition, the differential TNF sensitization observed across the tested cancer models warrants further investigation of combination strategies with TNF-inducing agents and other anticancer therapies. As the present study is based on cell lines, immune-mediated contributions to compound activity could not be assessed, and evaluation in immunocompetent models will be required.

Together, these studies will guide the rational optimization of the benzodiazepinone scaffold toward compounds tailored to distinct IAP-associated tumor contexts and may support the development of new therapeutic approaches for aggressive and chemoresistant cancers.

## 4. Materials and Methods

### 4.1. Cell Culture and Cytotoxicity Assay

MDA-MB-231, BT-549, NCI-H1299, and PC-3 cells were purchased from (LGC Standards, Guildford, UK cat n° ATCC-CRL-5803) or gently provided by Dr. Lecis at the Istituto Nazionale dei Tumori (Milan, Italy) and cultured under recommended conditions. The cells were grown in RPMI-1640 medium supplemented with 10% heat-inactivated fetal calf serum, 1 mg·mL^−1^ penicillin, 100 μg·mL^−1^ streptomycin and 2 mM l-glutamine (all from Sigma-Aldrich Co., St Louis, MO, USA) at 37 °C in humidified air containing 5% CO_2_. Growth and morphology were monitored on a weekly basis.

All compounds were initially dissolved in DMSO at 25 mM. Fresh solutions were prepared for each experiment and diluted in culture medium containing 10% FCS to obtain the desired final concentrations. Cell viability was assessed using the PrestoBlue™ Cell Viability Reagent (Thermo Fisher Scientific, Rodano, Italy) based on the reduction of resazurin to the fluorescent metabolite resorufin by metabolically active cells. Cells were seeded in black-walled, clear-bottom 96-well microplates at a density of 5 × 10^4^ cells per well. An initial screening of **FC2** derivatives was performed at a fixed concentration (12.5 μM) to identify the most active compounds. The concentration of 12.5 µM was selected based on the previously reported EC_50_ value of the parental compound 4r in MDA-MB-231 cells (7.5 ± 1.7 µM) and was used for the preliminary screening of the new derivatives. Selected compounds were subsequently subjected to dose–response analysis over a concentration range of 0.8–100 μM, using DMSO as vehicle control, in the absence or presence of TNFα (50 ng/mL). After 48 h of treatment, fluorescence was measured using a microplate reader (Tristar2 S, Berthold Technologies, Bad Wildbad, Germany) with excitation and emission wavelengths set at 560 nm and 585 nm, respectively. Each experimental condition was performed in at least five replicates. The half-maximal effective concentration (EC_50_) was determined by plotting the percentage of cell survival as a function of compound concentration (A) and fitting the data to the following equation:

% survival = (T_max − T_min) × A/(A + EC_50_) + T_min

where T_max and T_min represent the maximum and minimum effects induced by the compounds, respectively. Data analysis was performed using Igor Pro 8.04 software (Lake Oswego, OR, USA).

### 4.2. General Description of the Synthesis of the Compounds

All solvents and chemicals used for synthesis and analysis were of reagent grade. If not explicitly indicated in the text, all other chemicals were provided by Merck (Milan, Italy), Sigma-Aldrich (St. Louis, MO, USA), and VWR (Radnor Township, PA, USA) used as received unless purified.

Where necessary, the solvent was removed using a rotary evaporator at a pressure of approximately 10–50 Torr.

All final products utilized in biological assays had a purity of 95% or higher based on the analytical HPLC/MS analysis. Compound purity was determined by integrating peak areas of the chromatogram obtained in the liquid phase, monitored at 254 nm.

A Phenomenex C18 Luna HPLC column (4.6 × 250 mm, 5 μm) (Torrance, CA, USA) was used for analytical separation in an Agilent 1260 high-performance liquid chromatograph (Agilent Technologies, Santa Clara, CA, USA). The purification of the products by preparative chromatography required an Agilent 1260 Infinity HPLC with a Phenomenex C18 Luna (21.2 × 250 mm, 15 μm) column. Preparative HPLC consisted in the following gradient: from 0 to 5 min at 20% eluent B, from 5 min to 40 min at 100% eluent B, and from 40 to 45 min at 100% eluent B. Eluent A was water with 0.1% formic acid (FOA), and eluent B was acetonitrile with 0.1% FOA.

Liquid chromatography–electrospray mass spectrometry (HPLC-ESI-MS, Agilent Technologies, Santa Clara, CA, USA) was used to analyse the intermediates and the raw products using an Agilent 1100 series LC/MSD ion trap instrument.

The HRMS experiments were performed using a Q Exactive Orbitrap instrument by Thermo Scientific (Waltham, MA, USA).

The nuclear magnetic resonance (NMR) analyses were performed using a Jeol 400 MHz spectrometer (Jeol LTD, Akishima, Tokyo, Japan). The proton spectra and the carbon spectra were acquired at room temperature at 400 MHz and at 100 MHz, respectively. Chemical shifts were reported in units (ppm) relative to tetramethylsilane (TMS) as an internal standard. Coupling constants (J), when reported, are expressed in Hertz (Hz).

The compounds were dissolved in dimethyl sulphoxide (DMSO) to a stock solution of 100 mM and were further diluted with phosphate-buffered saline (PBS) for cell treatment.

Synthetic procedures and compound characterization are provided in the Supplementary Materials.

## Figures and Tables

**Table 1:** EC_50_ on MDA-MB-231 and NCI-H1299 of benzodiazepinone derivatives in the absence/presence of 50 ng/mL TNFα. EC_50_ values were calculated after three independent experiments in MDA-MB-231 and NCI-H1299 cells.

				MDA-MB-231		NCI-H1299	
	R_1_		R_2_	EC_50_ (−/+ TNFα) [mM]		EC_50_ (−/+ TNFα) [mM]	
**4r**	phenyl -	OH, OCH_3_ (*para*, *meta*)	benzoyl	7.5 ± 1.7	12.9 ± 1.9	2.1 ± 0.4	2.2 ± 0.5
**1**	phenyl -	OCH_3_ (*meta*, *para*, *meta*)	benzoyl	5.0 ± 1.0	13.4 ± 2.6	42.8 ± 1.9	52.5 ± 6.1
**2**	CH_2_-CH_2_	- phenyl	benzoyl	10.5 ± 1.9	11.5 ± 1.9	47.4 ± 2.8	31.0 ± 6.7
**3**	phenyl -	Br (*meta*)	benzoyl	7.1 ± 2.9	7.1 ± 1.7	1.3 ± 0.4	1.2 ± 0.3
**4**	phenyl -	Cl (*meta*)	benzoyl	9.3 ± 1.1	4.5 ± 1.0	1.3 ± 0.3	1.3 ± 0.3
**5**	phenyl -	Br, OH (*meta*, *para*)	benzoyl	7.4 ± 2.3	10.6 ±1.6	2.9 ± 0.7	1.8 ± 0.8
**6**	phenyl -	F (*meta*)	benzoyl	5.4 ± 0.8	9.2 ± 1.9	1.2 ± 0.5	1.3 ± 0.3
**10a**	phenyl -	OH, OCH_3_ (*para*, *meta*)	benzoyl, F (*para*)	16.7 ± 2.5	4.6 ± 0.8	1.1 ± 0.2	1.5 ± 0.4
**10b**	phenyl -	OH, OCH_3_ (*para*, *meta*)	benzoyl, CH_3_ (*para*)	17.0 ± 2.8	8.2 ± 1.3	1.8 ± 0.5	1.3 ± 0.7

For the structure of the complete scaffold, refer to Figure 1.

## Data Availability

The data generated and analyzed during this study are available from the corresponding author upon request.

## Supplementary Materials

The following supporting information can be downloaded at https://www.mdpi.com/article/10.3390/molecules31214016/s1, S1: Synthesis of derivatives with variations in the R_1_ group; S2: synthesis of derivatives with variations in the R_2_ group; S3: ^1^H NMR and ^13^C NMR spectra of compounds **5**, **6** and **10a**.

## Abbreviations

The following abbreviations are used in this manuscript:

BIR	Baculoviral IAP Repeat
cIAP	Cellular IAP
EGFR/Snai2	Epidermal Growth Factor Receptor/Snail family transcriptional repressor 2
EMT	Epithelial–Mesenchymal Transition
IAP	Inhibitor of Apoptosis Protein
IBM	IAP Binding Motif
IκBα	Nuclear factor of kappa light polypeptide gene enhancer in B-cells inhibitor, alpha
NF-κB	Nuclear Factor kappa-light-chain-enhancer of activated B cells
PKC	Protein Kinase C
SM	Smac-Mimetic
Smac/DIABLO	Second Mitochondria-derived Activator of Caspases/Direct IAP Binding protein with LOw pI
TAB 1	TGF-beta Activated Kinase 1 (MAP3K7) Binding Protein 1
TNBC	Triple Negative Breast Cancer
TNFα	Tumor Necrosis Factor
TRAF2	Tumor necrosis factor Receptor-Associated Factor 2
XIAP	X chromosome-linked IAP
