# Supplementary material for: Tumor-Dependent Cytotoxicity of Novel Benzodiazepinones Across Cancers with Distinct IAP-Associated Aggressiveness
Source: Molecules. 2026 Nov 7;31(21):4016. doi: 10.3390/molecules31214016 (PMC13765466; doi:10.3390/molecules31214016)
Supplement: Supplementary file 1 [file molecules-31-04016-s001.zip › molecules-4654409-supplementary.pdf]

Supporting Information

Tumor-dependent cytotoxicity of novel benzodiazepinones across cancers with distinct IAP-associated aggressiveness

Michele Fiore[1a†], Dario Lunaccio[2a†], Elisa Fagnani[1b], Paolo Cocomazzi[1b], Francesco Bonì[1b], Bruno Tasso[2b], Annalisa Salis[2a], Enrico Millo[2a*], Federica Cossu[1b*]

[1] Institute of Biophysics-National Research Council (IBF-CNR) Genoa Unit, via De Marini, 6, 16149 Genova, Italy; michele.fiore@ibf.cnr.it (M.F)

[2] Department of Experimental Medicine, Section of Biochemistry, University of Genoa, Viale Benedetto XV 1, 16132 Genoa, Italy; lunacciodario@gmail.com (D.L.); annalisa.salis@unige.it (A.S.)

[3] Institute of Biophysics-National Research Council (IBF-CNR) Milan Unit, Via Corti 12, I-20133 Milano, Italy; elisafagnani@cnr.it (E.F.); paologiuseppecocomazzi@cnr.it (P.C.); francesco.boni@cnr.it (F.B.)

[4] Department of Pharmacy, Section of Medicinal Chemistry, School of Medical and Pharmaceutical Sciences, University of Genoa, Viale Benedetto XV, 3, 16132 Genoa, Italy; bruno.tasso@unige.it (B.T.)

* Correspondence: enrico.millo@unige.it (E.M.); federica.cossu@cnr.it (F.C.); Tel.: +39-0223699541 (F.C.); +39-010-335-3032 (E.M.)

† These authors contributed equally to this work.

The *Supporting Information concern:*

1. *Synthesis of derivatives with variations in the R_1 group*
2. *Synthesis of derivatives with variations in the R_2 group*
3. *^1H NMR and ^{13}C NMR spectra of compounds **5**, **6** and **10a***

S1. Synthesis of derivatives with variations in the R_1 group

To synthesize the different benzodiazepine derivatives in terms of the R_1 substituent, the following procedure was adapted from the previous protocol published (Fiore, 2023).

The synthesis of 1H-dibenzo[1,4]diazepin-1-one derivatives (**1-6**) was achieved through a multi-step condensation process. Initially, 3,4-diaminobenzophenone (212.2 mg, 1 mmol) reacted with 1,3 cyclohexanedione (112.1 mg, 1 mmol) in a 1:1 mixture of H_2O and EtOH at T= 80 °C. After a three-hour stirring period, the resulting enamine regioisomers were isolated via preparative HPLC. The peak of interest was concentrated and lyophilized to obtain 3-((2-amino-5-benzoylphenyl)amino)cyclohex-2-en-1-one as a brown oil (199.1 mg, 65%). Then, this intermediate (36.8 mg, 0.12 mmol) underwent a subsequent condensation with

various aldehydes (0.12 mmol) for 24 hours, catalysed by a few drops of acetic acid, to yield the final products. Following purification, the target compounds were obtained in moderate yields.

7-benzoyl-11-(3,4,5-trimethoxyphenyl)-2,3,4,5,10,11-hexahydro-1H-dibenzo[b,e][1,4]-diazepin-1-one (**1**)

The title compound was obtained by a condensation reaction between a solution of 3-((2-amino-5-benzoylphenyl)amino)cyclohex-2-en-1-one (36.8 mg, 0.12 mmol) and 3,4,5-trimethoxybenzaldehyde (23.5 mg, 0.12 mmol) in EtOH. The product, which precipitated as a yellowish powder from the crude reaction mixture, was subsequently purified after washing with ethanol at T=4 °C (11.6 mg, yield 20%).

^{1}H NMR (400 MHz, DMSO-d_6) δ 8.98 (s, 1H, NH), 7.63 – 7.50 (m, 3H, arom), 7.47 – 7.39 (m, 3H, arom), 7.01 – 6.84 (m, 4H, arom), 6.59 (d, J = 8.0 Hz, 1H, CH), 5.65 (d, J = 5.9 Hz, 1H, NH), 3.67 (s, 3H, OCH_3), 3.62 (s, 3H, OCH_3), 3.60 (s, 3H, OCH_3),2.77 – 2.52 (m, 2H, CH_2), 2.30 – 2.16 (m, 2H, CH_2), 1.98 – 1.75 (m, 2H, CH_2).

^{13}C NMR (101 MHz, DMSO-d_6) δ 193.67, 192,13, 157.38, 145.67, 145.01, 143.90, 138.70, 132.24, 130.89, 130.38, 130.25, 129.56, 128.84, 128.15, 127.61, 125.78, 123.57, 122.85, 120.06, 114.35, 113.61, 111.56, 59.89, 56.16, 56.13, 54.88, 36.39, 31.11, 21.59.

The HRMS (ESI) was calculated for $C_{29}H_{29}N_2O_5$ $[M+H]^+$ 485,20782 found 485,20758

7-benzoyl-11-(3-phenylpropyl)-2,3,4,5,10,11-hexahydro-1H-dibenzo[b,e][1,4]diazepin-1-one (**2**)

The title compound was obtained by condensation reaction between a solution of intermediate described in the general procedure above and 4-phenylpropanal (15.8 μL, 0.12 mmol) in EtOH. After 24h, the reaction mixture was diluted with dichloromethane (3 mL) and washed with water (3 x 3 mL). The organic phase was dried over Na_2SO_4, concentrated under reduced pressure and purified by preparative HPLC. The peak of interest was concentrated to obtain the title compound (11.5 mg, 22%) as yellowish solid with purity of >95% as determined by HPLC-MS.

^{1}H NMR (400 MHz, DMSO-d_6) δ 8.97 (s, 1H, NH), 7.64 – 7.51 (m, 3H, arom), 7.48 – 7.41 (m, 3H, arom), 7.27 – 7.03 (m, 4H, arom), 6.98 – 6.83 (m, 3H, arom), 5.64 (d, *J* = 5.8 Hz, 1H, NH), 4.58 (m, 1H, CH), 2.81 – 2.52 (m, 4H, $2CH_2$), 2.31 – 2.15 (m, 2H, CH_2), 2.03 – 1.69 (m, 4H, $2CH_2$).

^{13}C NMR (101 MHz, DMSO-D_6) δ 193.74, 192.58, 156.37, 145.53, 144.92, 143.76, 138.70, 132.29, 130.55, 130.41, 130.22, 129.56, 128.78, 128.27, 127.63, 125.91, 123.57, 122.84, 120.06, 114.37, 113.62, 111.57, 54.87, 36.59, 36.41, 32.19, 31.12, 21.85.

The HRMS (ESI) was calculated for $C_{28}H_{27}N_2O_2$ $[M+H]^+$ 423,20743 found 423,20756

7-benzoyl-11-(3-bromophenyl)-2,3,4,5,10,11-hexahydro-1H-dibenzo[b,e][1,4]diazepin-1-one (**3**)

3-bromobenzaldehyde (22,2 mg, 0.12 mmol) was added to a solution in EtOH of intermediate described in the general procedure above. The product, which precipitated as a yellowish powder from the crude reaction mixture, was subsequently purified after washing with ethanol at T= 4 °C (25.6 mg, 45%).

^{1}H NMR (400 MHz, DMSO-d_6) δ 8.99 (s, 1H, NH), 7.62 – 7.52 (m, 3H, arom), 7.50 – 7.41 (m, 3H, arom), 7.19 (td, *J* = 8.0, 5.8 Hz, 1H, arom), 7.13 – 7.04 (m, 2H, arom), 6.97 – 6.83 (m, 3H, arom), 6.69 (d, *J* = 8.2 Hz, 1H, CH), 5.65 (d, *J* = 5.9 Hz, 1H, NH), 2.79 – 2.53 (m, 2H, CH_2), 2.30 – 2.16 (m, 2H, CH_2), 1.98 – 1.77 (m, 2H, CH_2).

^{13}C NMR (101 MHz, DMSO-D_6) δ 193.17, 192.2026, 157.38, 145.87, 145.11, 143.74, 138.70, 132.24, 130.52, 130.41, 130.24, 129.58, 128.91, 128.36, 127.29, 125.83, 123.57, 123.01, 120.09, 114.37, 113.60, 111.62, 54.89, 36.39, 31.14, 21.87.

The HRMS (ESI) was calculated for $C_{26}H_{22}BrN_2O_2$ $[M+H]^+$ 473.08646 found 473.08637

7-benzoyl-11-(3-chlorophenyl)-2,3,4,5,10,11-hexahydro-1H-dibenzo[b,e][1,4]diazepin-1-one (**4**)

3-chlorobenzaldehyde (16.9 mg, 0.12 mmol) was added to a solution in EtOH of 3-((2-amino-5-benzoylphenyl)amino)cyclohex-2-en-1-one. The yellowish precipitate was purified by preparative HPLC (9.8 mg, 19%).

^{1}H NMR (400 MHz, DMSO-d_6) δ 9,02 (s, 1H, NH), 7.64 – 7.53 (m, 3H, arom), 7.49 – 7.42 (m, 3H, arom), 7.19 (td, *J* = 7.9, 6.0 Hz, 1H, arom), 7.14 – 7.05 (m, 2H, arom), 6.98 – 6.83 (m, 3H, arom), 6.68 (d, *J* = 8.2 Hz, 1H, CH), 5.69 (d, *J* = 5.9 Hz, 1H, NH), 2.78 – 2.53 (m, 2H, CH_2), 2.31 – 2.15 (m, 2H, CH_2), 1.98 – 1.78 (m, 2H, CH_2).

^{13}C NMR (101 MHz, DMSO-d_6) δ 193.27, 191.93, 157.45, 145.84, 144.96, 143.88, 138.71, 132.25, 130.53, 130.42, 130.24, 129.55, 128.89, 128.29, 127.63, 125.94, 123.58, 122.99, 120.05, 114.33, 113.59, 111.61, 54.88, 36.41, 31.12, 21.85.

The HRMS (ESI) was calculated for $C_{26}H_{22}ClN_2O_2$ $[M+H]^+$ 429.13697 found 429.13663

7-benzoyl-11-(3-bromo-4-hydroxyphenyl)-2,3,4,5,10,11-hexahydro-1H-dibenzo[b,e][1,4]diazepin-1-one (**5**)

The title compound was obtained by condensation reaction between a solution of intermediate described in the general procedure above and 3-bromo-4-hydroxybenzaldehyde (24.1 mg, 0.12 mmol) in EtOH. The yellowish powder was purified by crystallization in acetonitrile (14.0 mg, 24%).

^{1}H NMR (400 MHz, DMSO-d_6) δ 9.95 (s, 1H, OH), 8.95 (s, 1H, NH), 7.61 – 7.41 (m, 6H, arom), 7.23 (d, *J* = 2.1 Hz, 1H, arom), 7.10 (dd, *J* = 8.3, 1.9 Hz, 1H, arom), 6.98 (d, *J* = 6.1 Hz, 1H, arom), 6.85 (dd, *J* = 8.5, 2.2 Hz, 1H,

arom), 6.71 – 6.62 (m, 2H, arom + CH), 5.63 (d, *J* = 6.0 Hz, 1H, NH), 2.73 – 2.57 (m, 2H, CH_2), 2.29 – 2.17 (m, 2H, CH_2), 1.98 – 1.79 (m, 2H, CH_2).

^{13}C NMR (101 MHz, DMSO-d_6) δ 194.29, 193.13, 157.60, 152.78, 144.14, 138.77, 137.00, 132.19, 132.15, 130.33, 129.56, 128.88, 128.17, 127.66, 125.90, 122.91, 120.04, 118.65, 116.35, 111.97, 109.44, 54.36, 36.46, 31.07, 21.93.

The HRMS (ESI) was calculated for $C_{26}H_{22}BrN_2O_3$ $[M+H]^+$ 489.08137 found 489.08100

11-(4-hydroxy-3-methoxyphenyl)-7-(4-methylbenzoyl)-2,3,4,5,10,11-hexahydro-1H-dibenzo[b,e][1,4]diazepin-1-one (**6**)

The title compound was obtained by condensation reaction between a solution of intermediate described in the general procedure above and 3-fluoro-benzaldehyde (14.9 mg, 0.12 mmol) in EtOH . The yellowish powder was purified by preparative HPLC (9.0 mg, 18%).

^1H NMR (400 MHz, DMSO-d_6) δ 8.99 (s, 1H, NH), 7.61 – 7.52 (m, 3H, arom), 7.52 – 7.43 (m, 3H, arom), 7.18 (td, *J* = 7.9, 6.2 Hz, 1H, arom), 7.13 – 7.06 (m, 2H, arom), 6.96 – 6.81 (m, 3H, arom), 6.70 (d, *J* = 8.3 Hz, 1H, CH), 5.75 (d, *J* = 6.0 Hz, 1H, NH), 2.76 – 2.55 (m, 2H, CH_2), 2.30 – 2.16 (m, 2H, CH_2), 1.98 – 1.83 (m, 2H, CH_2).

^{13}C NMR (101 MHz, DMSO-d_6) δ 194.31, 193.26, 163.86, 161.44, 157.86, 147.86, 143.88, 138.71, 132.18, 130.53, 130.45, 130.24, 129.56, 128.89, 128.29, 125.94, 123.61, 123.59, 122.99, 120.05, 114.43, 113.71, 113.50, 111.61, 54.91, 36.41, 31.07, 21.87.

The HRMS (ESI)was calculated for $C_{26}H_{22}FN_2O_2$ $[M+H]^+$ 413.16652 found 413.16608

S2. Synthesis of derivatives with variations in the R_2 group

To a solution of phenylacetonitrile derivative (1 eq, **7a** or **7b**) and 5-chloro-2-nitroaniline (1 eq) in anhydrous DMF at T =0 °C, KOtBu (2 eq) was added, resulting in an intense dark blue mixture. The reaction was stirred at room temperature for 4 hours until completeness (monitored by HPLC) before being cooled again to T =0 °C. Hydrogen peroxide (30% aq. solution, 3.3 eq) was then introduced, and the mixture was stirred overnight while slowly reaching room temperature. Upon completion, the dark brown solution was poured into 1 N HCl to induce the formation of a bright orange precipitate. After 30 minutes of vigorous stirring, an orange solid corresponding to the (3-amino-4-nitrophenyl)(phenyl)methanone derivative (**8a** or **8b**) was isolated by filtration and dried in vacuo at T=30 °C for 12 hours.

To a stirred solution of (3-amino-4-nitrophenyl)(phenyl)methanone derivative (1eq) in 5 mL ethanol, $SnCl_2x2H_2O$ (6eq) was added. The reaction was maintained at T =75 °C under N_2 for 3.5 hours. After cooling,

the mixture was basified to pH 9–10 (50% NaOH) and vacuum filtered. The solvent was removed in vacuo, followed by extraction of the residue with ethyl acetate. The organic layer was washed with brine, dried with anhydrous Na_2SO_4, and concentrated to provide (3,4-diaminophenyl)methanone derivative **9a** and **9b** (yield 86% for **9a** and 75% for **9b**) as an orange solid. The crude product was obtained as orange crystals via recrystallization from EtOH and used immediately in the next step without any further purification.

9a (1 eq) and **9b (**1 eq) were respectively conjugated with 1,3 cyclohexanedione (1eq) in a 1:1 mixture of water and EtOH at T=80 °C according to procedure for R_1 benzodiazepine derivative synthesis to obtain 3-((2-amino-5-benzoylphenyl)amino)cyclohex-2-en-1-one derivatives.

Finally, 3-((2-amino-5-benzoylphenyl)amino)cyclohex-2-enone derivative (1 eq, 0.12 mmol) was conjugated with various aldehydes (1 eq, 0.12 mmol) for 24 hours, catalysed by a few drops of acetic acid, to yield the final products **10a** and **10b**. Following purification, the target compounds were obtained in moderate yields.

7-(4-fluorobenzoyl)-11-(4-hydroxy-3-methoxyphenyl)-2,3,4,5,10,11-hexahydro-1H-dibenzo[b,e][1,4]diazepin-1-one (**10a**)

The title compound was obtained with the condensation between the 3-((2-amino-5-(4-fluorobenzoyl)phenyl)amino)cyclohex-2-enone **9a** (38.9 mg, 0.12 mmol) and 4-hydroxy-3-methoxybenzaldehyde (18.9 mg, 0.12 mmol) in EtOH. The title compound **10a** was obtained as a yellowish powder after preparative HPLC purification (9.6 mg, 22%).

^{1}H NMR (400 MHz, DMSO-*d*$_6$) δ 8.88 (s, 1H, NH), 8.67 (s, 1H, OH), 7.67 – 7.57 (m, 2H, arom), 7.43 (d, *J* = 2.0 Hz, 1H, arom), 7.35 – 7.25 (m, 2H, arom), 7.06 (dd, *J* = 8.3, 2.0 Hz, 1H, arom), 6.99 (d, *J* = 6.2 Hz, 1H, arom), 6.81 (d, *J* = 2.0 Hz, 1H, arom), 6.69 (d, *J* = 8.3 Hz, 1H, CH), 6.46 (d, *J* = 8.1 Hz, 1H, arom), 6.37 (dd, *J* = 8.2, 2.0 Hz, 1H, arom), 5.66 (d, *J* = 5.9 Hz, 1H, NH), 3.60 (s, 3H, OCH_3), 2.72 – 2.57 (m, 2H, CH_2), 2.29 – 2.18 (m, 2H, CH_2), 1.98 – 1.78 (m, 2H, CH_2).

^{13}C NMR (101 MHz, DMSO-*d*$_6$) δ 193.11, 192.93, 163.29, 157.41, 147.73, 145.30, 144.69, 135.60, 135.35, 135.32, 132.38, 132.29, 130.51, 127.81, 125.71, 122.67, 120.03, 119.49, 115.98, 115.76, 115.16, 112.51, 112.34, 55.90, 54.87, 36.54, 31.11, 21.98.

The HRMS (ESI) was calculated for $C_{27}H_{24}FN_2O_4$ $[M+H]^+$ 459.17200 found 459.17184

11-(4-hydroxy-3-methoxyphenyl)-7-(4-methylbenzoyl)-2,3,4,5,10,11-hexahydro-1H-dibenzo[b,e][1,4]diazepin-1-one (**10b**)

The title compound was obtained with the condensation between the 3-((2-amino-5-(4-methylbenzoyl)phenyl)amino)cyclohex-2-enone **9b** (39.9 mg, 0.12 mmol) and 4-hydroxy-3-methoxybenzaldehyde (18.9 mg, 0.12 mmol) in ethanol. The title compound **10b** was obtained as a yellowish powder after preparative HPLC purification (14.2 mg, 25%).

^{1}H NMR (400 MHz, DMSO-d_6) δ 8.89 (s, 1H, NH), 8.73 (s, 1H, OH), 7.69 – 7.58 (m, 2H, arom), 7.36 – 7.25 (m, 2H, arom), 7.08 – 6.97 (m, 3H, arom), 6.80 (d, *J* = 2.0 Hz, 1H, arom), 6.67 (d, *J* = 8.3 Hz, 1H, CH), 6.48 -6.39 (m, 2H, arom), 5.67 (d, *J* = 6.0 Hz, 1H, NH), 3.61 (s, 3H, OCH_3), 2.71 – 2.58 (m, 2H, CH_2), 2.36 (s, 3H, CH_3), 2.30 – 2.17 (m, 2H, CH_2), 1.98 – 1.76 (m, 2H, CH_2).

^{13}C NMR (101 MHz, DMSO-d_6) δ 192.98, 191.76, 162.97, 157.08, 147.65, 145.18, 144.66, 142.24, 135.62, 135.38, 134.77, 132.29, 130.49, 127.63, 125.70, 122.59, 120.14, 119.48, 116.02, 115.14, 112.49, 112.33, 55.89, 54.82, 36.55, 31.12, 21.91, 21.36.

The HRMS (ESI) was calculated for $C_{28}H_{27}N_2O_4$ $[M+H]^+$ 455,19726 found 455,19765

S3. *^{1}H NMR and ^{13}C NMR spectra of compounds 5, 6 and 10a*

^{1}H - Compound 5

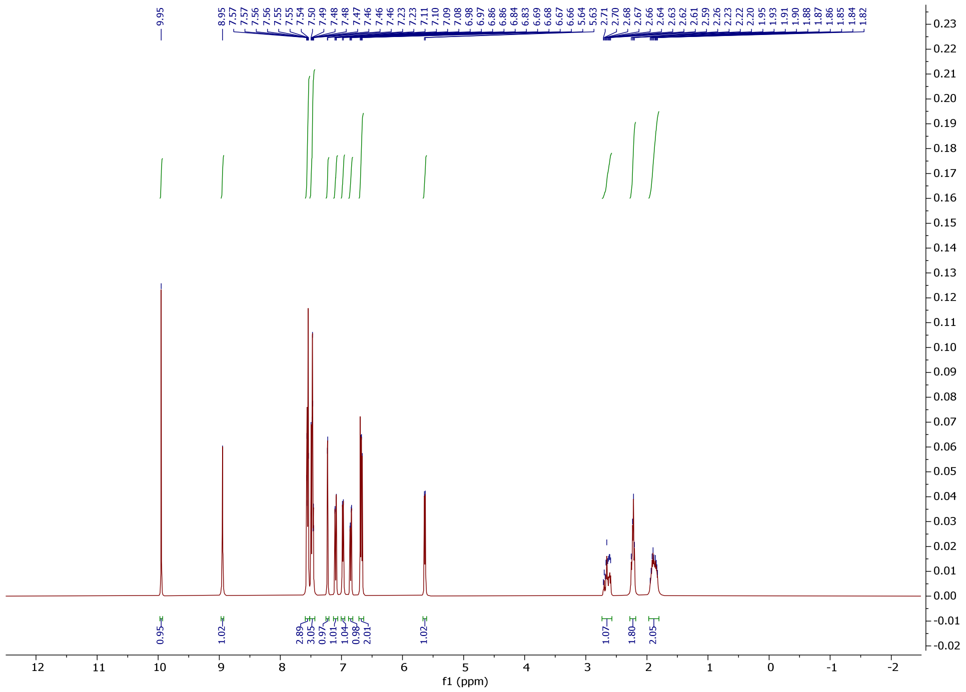

^{13}C - Compound 5

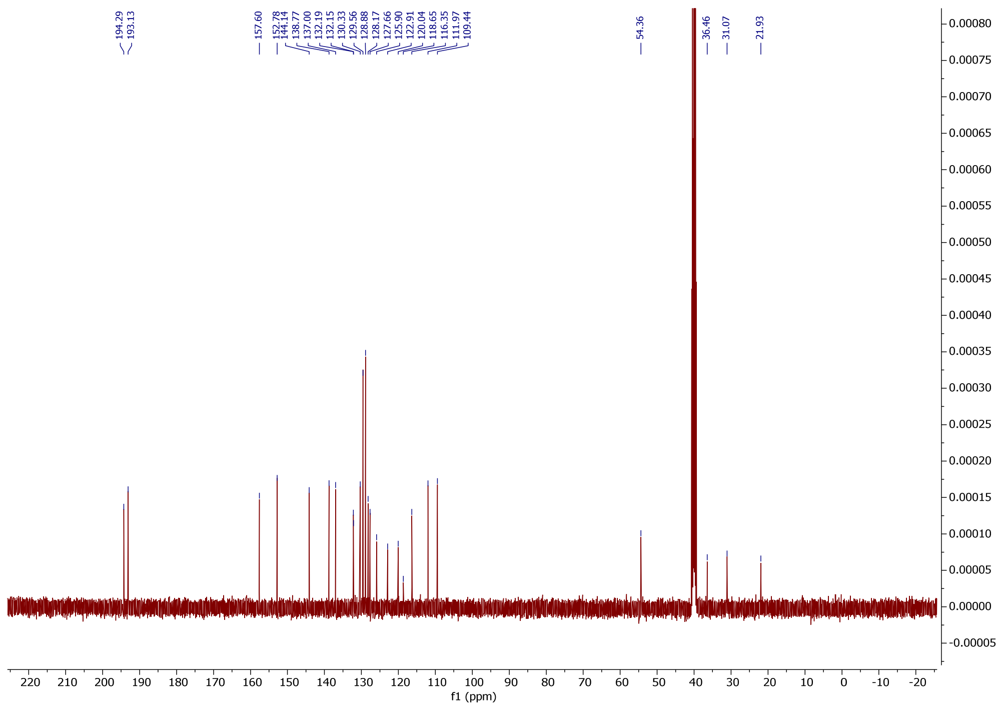

^{1}H - Compound 6

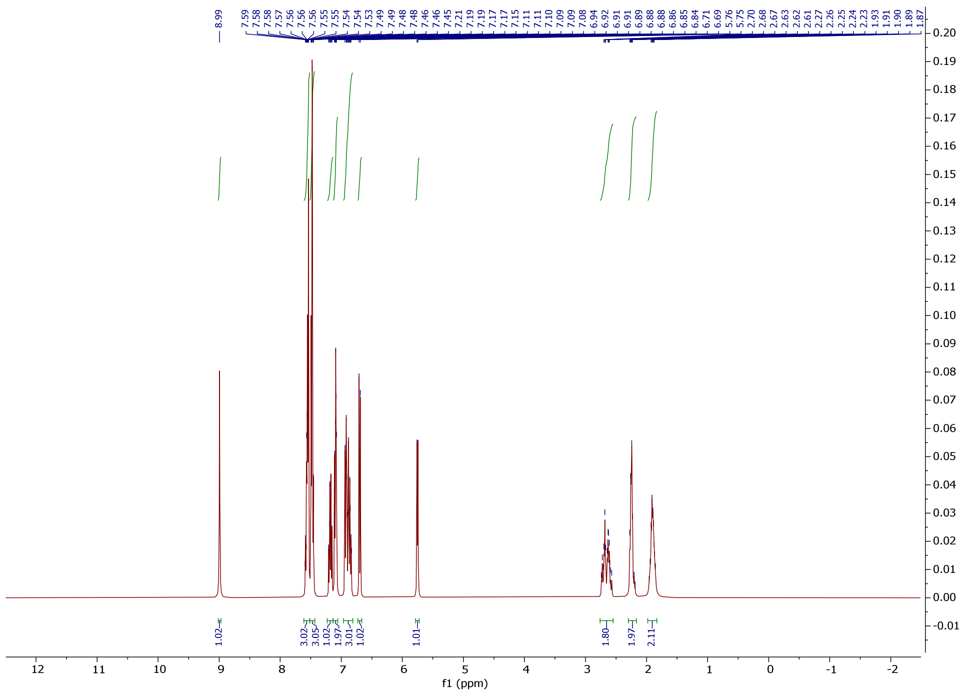

^{13}C - Compound 6

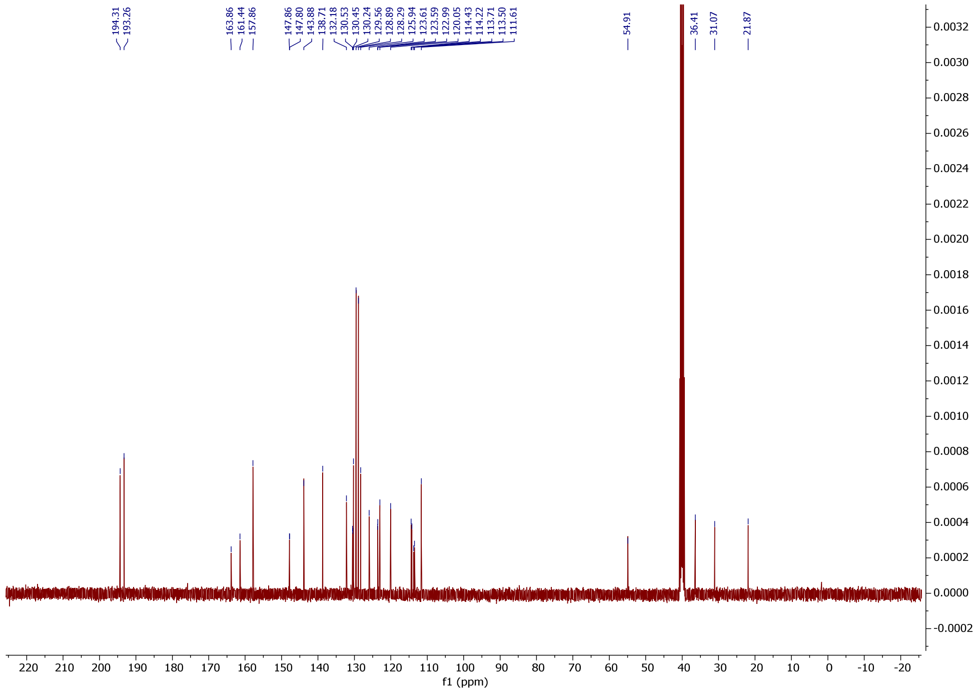

¹H - Compound 10a

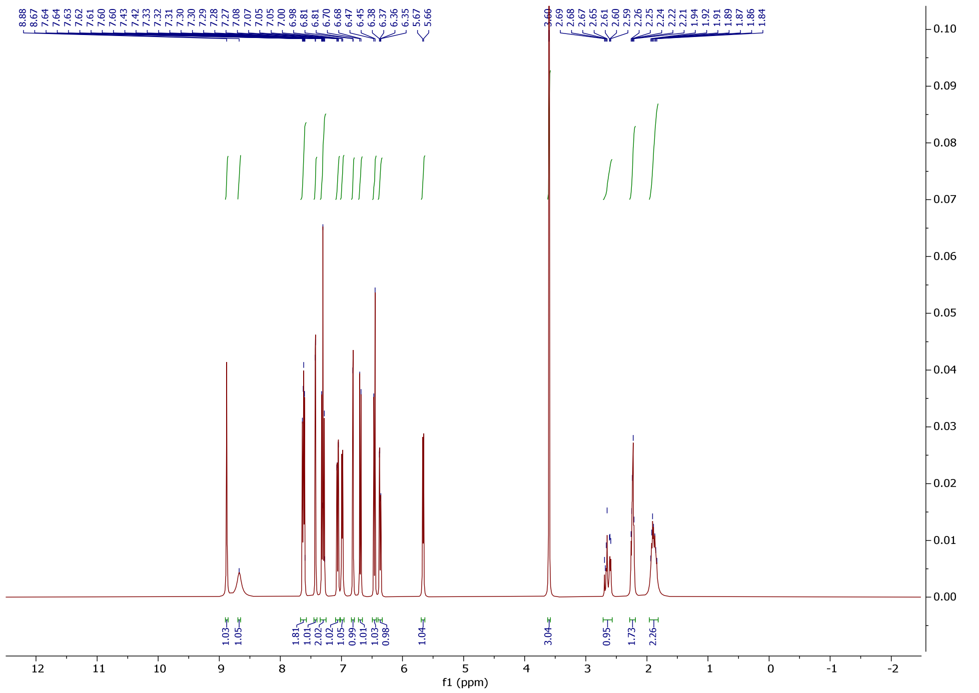

¹³C - Compound 10a

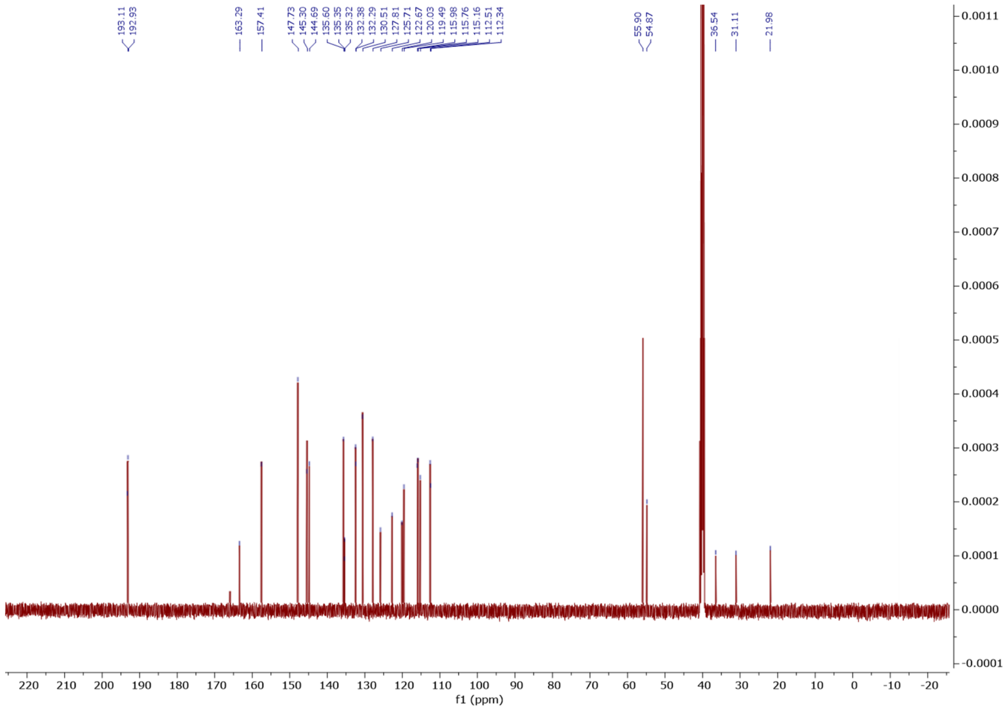
193.11
192.93
163.29
157.41
147.73
145.30
144.69
135.60
135.35
135.32
132.38
132.29
130.51
127.81
125.71
122.67
120.03
119.49
115.98
115.76
115.16
112.51
112.34
55.90
54.87
36.54
31.11
21.98
f1 (ppm)
